# Unusual case of parietal metastasis from papillary thyroid carcinoma: a case report

**DOI:** 10.11604/pamj.2022.42.176.34823

**Published:** 2022-07-05

**Authors:** Evrard Niyonkuru, Zineb Sami, Fadwa Bouyalik, Samira Benayad, Mohamed El Karroumi, Mehdi Karkouri

**Affiliations:** 1 Pathology Department, Ibn Rochd University Hospital Center, Casablanca, Morocco,; 2 Faculty of Medicine and Pharmacy, Hassan II University of Casablanca, Pathology Department, Ibn Rochd University Hospital Center, Casablanca, Morocco,; 3 Mohammed VI Center for Cancer Treatment, Ibn Rochd University Hospital Center, Casablanca, Morocco

**Keywords:** Papillary thyroid carcinoma, metastasis, skin, case report

## Abstract

Parietal metastasis is a very rare secondary location of papillary thyroid carcinoma. It associated with poor prognosis. We report a case of a 61-year-old woman with parietal metastasis from papillary thyroid carcinoma. The patient presented a parietal nodule on the back. In her past history, she had been diagnosed papillary thyroid carcinoma after total thyroidectomy and also reoperated for local recurrence. The CT scan performed has revealed metastasis to the lungs, bones, lymph nodes and adrenal glands. The parietal nodule was excised and submitted for histopathological examination. The histologic and immunohistochemical findings confirmed the thyroid origin. Although papillary thyroid carcinoma is a relatively indolent tumour, it can exhibit an unusual metastatic behaviour.

## Introduction

Papillary thyroid carcinoma is the most common malignant neoplasm arising in the thyroid gland and represents approximately 80% of all thyroid cancers [1]. It is an indolent malignancy with excellent prognosis and has a minimal potential for distant metastasis [2]. Skin metastasis is an unusual location and constitutes an ominous prognostic indicator [3]. We report a case of a patient suffering from papillary thyroid carcinoma with disseminated metastases.

## Patient and observation

**Figure 1:**
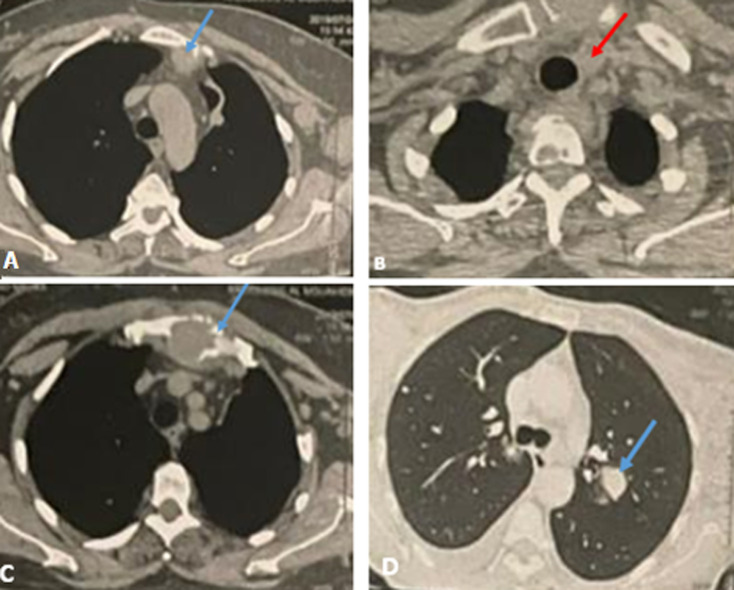
chest CT scan, mediastinal window (A, B, C), axial slices (A, B) showing adenopathies of the prevascular territory (blue arrow) and upper left laterotracheal territory (red arrow), osteolytic lesion of the sternum (C) (blue arrow), parenchymal window, axial slice showing a left apex dense nodule (blue arrow) (D)

**Patient information:** a 61-year-old woman presented with parietal nodule on the back, progressing gradually. Four years before, she had been diagnosed papillary thyroid carcinoma after total thyroidectomy. Two years after thyroidectomy, the tumour recurred locally and an excision has been performed. Afterwards, she developed a thoracic pain, motivating the realization of CT scan. The CT scan revealed multifocal metastatic bone lesions, cervical, axillary and mediastinal metastatic lymphadenopathies, metastatic nodules in the lungs and adrenal glands (Figure 1). The axillary adenectomy was performed following two axillary masses that histologically showed carcinomatous proliferation of papillary architecture in which the tumour cells were stained by thyroglobulin antibody confirming the thyroid origin. The evolution has been marked by the emergence of a parietal nodule on the back soft tissue.

**Clinical findings:** on physical examination, there was a well-limited, painless, firm and non-mobile nodule located in the back.

**Figure 2:**
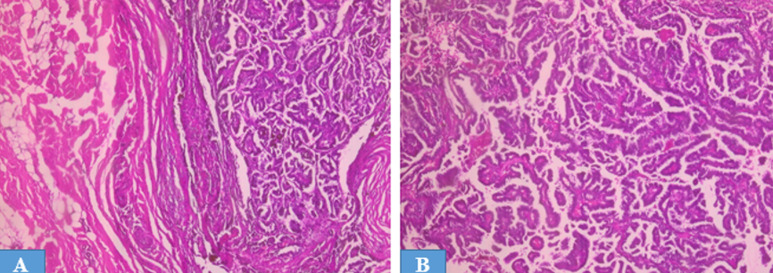
histological examination, hematoxylin and eosin stain, (A, 10x) and (B, 20x) showing a carcinomatous proliferation of papillary architecture

**Figure 3:**
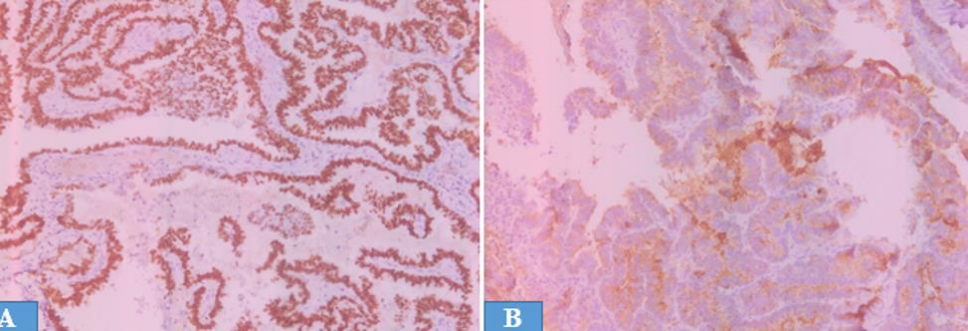
immunohistochemistry, the tumour cells showing positive staining for thyroid transcription factor-1 (A, 20x) and thyroglobulin (B, 20x)

**Diagnosis:** the nodule was excised and submitted for histopathological examination. The histological examination of the nodule revealed carcinomatous proliferation of papillary architecture with papillary-like nuclear features and psammomatous calcifications (Figure 2). Immunohistochemically, the tumour cells showed positive staining for thyroid transcription factor-1 and thyroglobulin (Figure 3). These findings supported the diagnosis of parietal metastasis from papillary thyroid carcinoma.

**Therapeutic interventions:** the patient also received radioiodine therapy and external radiotherapy.

**Follow-up and outcome of interventions:** the patient died of respiratory distress because of pulmonary metastases 1 month after the nodule excision.

**Informed consent:** the consent was obtained from the patient´s family.

## Discussion

Papillary thyroid carcinoma is one of histologic subtypes of well-differentiated thyroid cancers. The histologic subtype impacts the incidence and the site of metastatic spread [2]. Papillary thyroid carcinoma usually spreads by lymphatic routes to regional lymphatic nodes, however, invasion by vascular routes is uncommon [4]. Distant hematogenous metastases from papillary thyroid carcinoma occur in less than 10 % of patients [5]. The most common distant metastatic sites are bone, lungs and brain. Uncommon metastatic sites are liver, adrenal glands, kidney, pancreas, stomach, oesophagus, distant skeletal muscle, penis, eye, choroid, skin and submandibular gland [6]. The reported average age in well-differentiated thyroid cancer patients with unusual metastases is 68 years [7]. Our patient was 61 years old and developed metastases in the bones, lungs, lymph nodes, adrenal glands and the skin. To our knowledge, more than three metastatic sites occurring in the same patient have rarely been reported in large studies or case reports of well-differentiated thyroid cancer metastases, making our case exceptional. The present case had two uncommon metastatic sites, adrenal glands and skin. Batawil [8] has reported adrenal metastasis of papillary thyroid carcinoma with lung and bone metastases, which joins our result in the present case.

Skin metastasis occurs in the setting of disseminated neoplastic disease. The average time between the diagnosis of thyroid cancer and skin metastasis was 4.3 years according Dahl *et al*. study about metastatic thyroid carcinoma to the skin [3]. Skin metastases have been mostly described in the scalp, the face, the neck, and the upper thorax [9]. Our patient has developed skin metastasis in the back, 4 years after the initial diagnosis of papillary thyroid carcinoma. Skin metastasis of papillary thyroid carcinoma was confirmed after ruling out the different differential diagnoses. The main differential diagnoses are primary adnexal skin tumors, such as hidradenoma papilliferum and syringocystadenoma papilliferum and metastatic carcinomas from others sites like lung, ovary and stomach [3,10]. The clinical history, microscopic morphology features and immunohistochemical study allowed us to determine the thyroid origin as the same for other cases reported in the literature [3,9,11]. Uncommon metastatic sites of papillary thyroid carcinoma exhibit a poor prognosis especially skin location [3]. The death of the patient with skin metastasis usually occurs in 3 to 6 months [9]. The age of the patient at discovery of distant metastases impacts the prognosis. Durante and al have reported the 10-year survival rate at 95% in patients younger than 40 years old and 14% in patients older than 40 years [5].

## Conclusion

Even if papillary thyroid carcinoma is biologically considered as indolent tumour, it can display an aggressive behaviour and give distant metastases. Skin metastasis is an unexpected situation with a poor prognosis and usually occurs in advanced stage of disease.
